# Considerations for the use of Cre recombinase for conditional gene deletion in the mouse lens

**DOI:** 10.1186/s40246-019-0192-8

**Published:** 2019-02-15

**Authors:** Phuong T. Lam, Stephanie L. Padula, Thanh V. Hoang, Justin E. Poth, Lin Liu, Chun Liang, Adam S. LeFever, Lindsay M. Wallace, Ruth Ashery-Padan, Penny K. Riggs, Jordan E. Shields, Ohad Shaham, Sheldon Rowan, Nadean L. Brown, Tom Glaser, Michael L. Robinson

**Affiliations:** 1 Department of Biology, Miami University, Oxford, OH 45056 USA; 2 Nuclear Medicine Department, University of Cincinnati Medical Center, 234 Goodman Street, Cincinnati, OH 45219 USA; 3 Center for Gene Therapy, The Research Institute at Nationwide Children’s Hospital, Columbus, OH 43205 USA; 4 Department of Human Molecular Genetics and Biochemistry, Sackler Faculty of Medicine, Sagol School of Neurosciences, Tel Aviv University, 69978 Tel Aviv, Israel; 5 Department of Animal Sciences, Texas A&M University, College Station, TX 77843-2471 USA; 6 Department of Ophthalmology, Tufts University School of Medicine, Boston, MA 02111 USA; 7 Department of Cell Biology and Human Anatomy, University of California, Davis One Shields Avenue, Davis, CA 95616 USA; 8 Present Address: Solomon H. Snyder Department of Neuroscience, Johns Hopkins University School of Medicine, Baltimore, MD 21205 USA; 9 Present Address: Emory Children’s Center, Room 410, 2015 Uppergate Drive, Atlanta, GA 30322 USA

**Keywords:** Cre recombinase, Lens development, Transgenic mice

## Abstract

**Background:**

Despite a number of different transgenes that can mediate DNA deletion in the developing lens, each has unique features that can make a given transgenic line more or less appropriate for particular studies. The purpose of this work encompasses both a review of transgenes that lead to the expression of Cre recombinase in the lens and a comparative analysis of currently available transgenic lines with a particular emphasis on the *Le-Cre* and *P0-3.9GFPCre* lines that can mediate DNA deletion in the lens placode. Although both of these transgenes are driven by elements of the *Pax6* P0 promoter, the *Le-Cre* transgene consistently leads to ocular abnormalities in homozygous state and can lead to ocular defects on some genetic backgrounds when hemizygous.

**Result:**

Although both *P0-3.9GFPCre* and *Le-Cre* hemizygous transgenic mice undergo normal eye development on an *FVB/N* genetic background, *Le-Cre* homozygotes uniquely exhibit microphthalmia. Examination of the expression patterns of these two transgenes revealed similar expression in the developing eye and pancreas. However, lineage tracing revealed widespread non-ocular CRE reporter gene expression in the *P0-3.9GFPCre* transgenic mice that results from stochastic CRE expression in the *P0-3.9GFPCre* embryos prior to lens placode formation. Postnatal hemizygous *Le-Cre* transgenic lenses express higher levels of CRE transcript and protein than the hemizygous lenses of *P0-3.9GFPCre* mice. Transcriptome analysis revealed that *Le-Cre* hemizygous lenses deregulated the expression of 15 murine genes, several of which are associated with apoptosis. In contrast, *P0-3.9GFPCre* hemizygous lenses only deregulated two murine genes. No known PAX6-responsive genes or genes directly associated with lens differentiation were deregulated in the hemizygous *Le-Cre* lenses.

**Conclusions:**

Although *P0-3.9GFPCre* transgenic mice appear free from ocular abnormalities, extensive non-ocular CRE expression represents a potential problem for conditional gene deletion studies using this transgene. The higher level of CRE expression in *Le-Cre* lenses versus *P0-3.9GFPCre* lenses may explain abnormal lens development in homozygous *Le-Cre* mice. Given the lack of deregulation of PAX6-responsive transcripts, we suggest that abnormal eye development in *Le-Cre* transgenic mice stems from CRE toxicity. Our studies reinforce the requirement for appropriate CRE-only expressing controls when using CRE as a driver of conditional gene targeting strategies.

**Electronic supplementary material:**

The online version of this article (10.1186/s40246-019-0192-8) contains supplementary material, which is available to authorized users.

## Background

Few breakthroughs have transformed the field of genetic engineering more than the discovery and exploitation of site-specific DNA recombinases. While gene mutations provide essential insight into normal gene function, early embryonic lethality often limits the information provided by a constitutive deletion (knockout) of essential, multifunctional genes. Solutions for this limitation came from the development of microorganism-derived, site-specific DNA recombinases. These recombinases include Cre (derived from P1 bacteriophage [1]), Flp (derived from *S. cerevisiae* [2]), and Dre (derived from D6 bacteriophage [3]). Cre, Flp, and Dre utilize 34 bp loxP, 34 bp Frt, or 32 bp rox recombination recognition sequences, respectively [4]. Each enzyme catalyzes recombination between two copies of their specific recognition sequence resulting in integration, deletion, translocation, or inversion of DNA, depending on the location and orientation of the recognition sequences [5]. Among these, Cre recombinase (CRE) remains the most widely used for mammalian genetic engineering. In fact, the Mouse Genome Informatics database (http://www.informatics.jax.org/) contains more than 2500 CRE transgenes and is annotated with information about expression pattern, current availability, and relevant publications.

Geneticists first demonstrated the ability of CRE to mediate DNA recombination in a living mammal using the lens as an experimental platform. In this pioneering experiment, CRE recombination activated the expression of the SV40 large tumor antigen (TAg), exclusively in the developing lenses of transgenic mice [6]. Specifically, the mouse αA-crystallin promoter drove transgenic CRE expression in the lens to catalyze the deletion of a loxP-flanked (floxed) transcriptional termination sequence that blocked TAg expression from a separate transgene. The resultant bi-transgenic mice carrying both the CRE transgene (*mαA-Cre*) and the TAg-bearing transgene uniformly developed lens tumors. Although mice never spontaneously develop lens tumors, the potency of the SV40 TAg for tumor formation meant that only a few lens cells in the bi-transgenic mice would have to undergo CRE-mediated recombination for tumor formation to occur. However, subsequent experiments demonstrated that CRE catalyzed DNA recombination in mice with very high efficiency, spurring the creation of hundreds of different transgenic mouse lines with inducible and/or tissue-specific CRE expression [7]. Insertion of loxP sites within introns usually has little or no effect on host gene expression. Hence, CRE revolutionized functional genomics by limiting the pattern and onset of gene deletion.

**Fig. 1:**
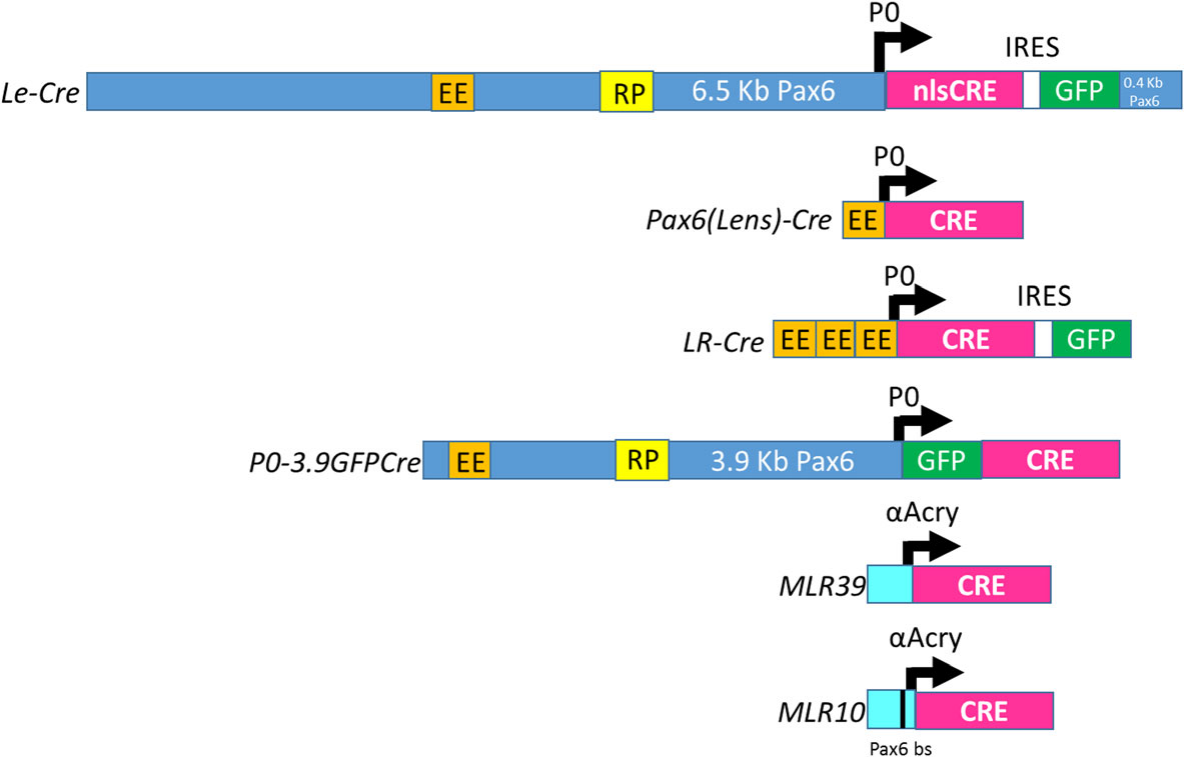
DNA constructs used to make Cre-expressing transgenic mouse lines. Black arrows represent the transcription start sites for the mouse *Pax6* P0 promoter (blue in the *Le-Cre* and *P0-3.9GFPCre* constructs) or the mouse αA-crystallin promoter (light blue in the *MLR39* and *MLR10* constructs). The pink and green boxes represent CRE and GFP coding sequences, respectively. The thin black line within the αA-crystallin promoter in the *MLR10* construct represents the engineered *Pax6* binding site. The orange EE and yellow RP boxes represent the *Pax6* ectodermal enhancer element and the retina/pancreas enhancer elements, respectively. The IRES (white box in the *Le-Cre* construct) stands for internal ribosome entry site, and nlsCre stands for Cre recombinase with an added nuclear localization sequence. There is approximately 400 bp of mouse *Pax6* sequence from the first intron included at the 3′ end of the *Le-Cre* transgenic construct, following the GFP coding sequence

Although *mαA-Cre* mice no longer exist, other CRE transgenes continue to facilitate studies of gene function in the developing lens (Fig. 1). *MLR39* and *MLR10* transgenes, like *mαA-Cre*, utilize the mouse αA-crystallin promoter to express CRE in developing lens fiber cells. In *MLR10*, an engineered insertion of a *Pax6* consensus-binding site within the transgenic αA-crystallin promoter drives transgene expression in the lens epithelium as well [8]. Several transgenes including *Le-Cre* [9], *Pax6(Lens)-Cre* [10], *LR-Cre* [11], and *P0-3.9GFPCre* [12], utilized different mouse *Pax6* P0 promoter/ectodermal enhancer sequences to drive CRE expression in the lens. Although designed to express CRE in neuronal and glial progenitor cells, the *Nes-Cre* transgenic mice made with the rat *Nestin* promoter/enhancer [13] is also active in the lens [14–16]. Of these transgenic mice, the most commonly used are (in order of use frequency to date) *Le-Cre* (62 publications), *MLR10* (28 publications), *MLR39*, (7 publications), *Nes-Cre* (3 publications), *P0-3.9GFPCre* (4 publications), and *LR-Cre* (3 publications). Like the *mαA-Cre* transgenics, the *Pax6(Lens)-Cre* mice [10] no longer exist. Each of these transgenes resulted from non-targeted DNA integrations into the genome following zygote microinjection.

The CRE tissue expression pattern represents the most important consideration to decide which of the available CRE transgenic lines will best suit any given project. However, the chromosomal location into which microinjected DNA constructs insert often affects transgene expression. These position effects frequently lead to unintended expression patterns or mosaic transgene expression [17–19]. Also, multiple tandem transgene copies, present in most transgenic lines produced by microinjection, may undergo epigenetic transcriptional silencing over time [20]. Despite these complications, each of the available CRE transgenic lines have distinct features that may provide unique advantages for some experimental purposes. Until now, no comparative analysis of CRE lines existed for lens gene deletion despite the usefulness of this information for future experimental designs.

CRE transgenes driven by P0 promoter/ectodermal enhancer of the mouse *Pax6* gene can mediate floxed gene deletion in the head surface ectoderm, which includes the lens placode, at embryonic day 9.0 (E9.0). In addition to catalyzing widespread deletion in the lens, these transgenes also result in floxed DNA deletion in precursors of the corneal epithelium, eyelid epithelium, conjunctiva, and surface ectoderm-derived ocular glands. The transgenic constructs used to produce the *Le-Cre* and *P0-3.9GFPCre* mice contain a large DNA fragment upstream of the murine *Pax6* P0 promoter, including the ectodermal enhancer as well as proximal and distal enhancers active in the endocrine pancreas [21–24]. As a result, both *Le-Cre* and *P0-3.9GFPCre* transgenic lines exhibit CRE expression in pancreatic precursors by E8.5, even earlier than in the presumptive lens ectoderm. Unlike the *Le-Cre* mice, the *P0-3.9GFPCre* mice expressed CRE in the apical ectodermal ridge in the limb buds and in the stomach mesenchyme [25]. The transgenic construct used to create the *Pax6(Lens)-Cre* mice contained only the 340 bp ectodermal enhancer and minimal *Pax6* P0 promoter, explaining the lack of pancreatic CRE expression in this mouse line [10]. Also, while transgene expression in the *Le-Cre and P0-3.9GFPCre* lines include both the lens placode and a swath of the head surface ectoderm extending to the olfactory epithelium, the *Pax6(Lens)-Cre* mice exhibited a more restricted, lens placode-proximal, expression pattern. Like the *Pax6(Lens)-Cre* transgene construct, the construct used to create the *LR-Cre* mice lacked the pancreatic *Pax6* enhancers, but this construct contained three tandem copies of the ectodermal enhancer cloned upstream of the *Pax6* P0 promoter. Transgene expression in the *LR-Cre* mice occurred in both the surface ectoderm encompassing the lens placode and in the optic vesicle at E9.5 [11]. Therefore, in contrast to the other CRE transgenes driven by the *Pax6* P0 promoter, the *LR-Cre* transgene catalyzes floxed DNA deletion in both the ocular surface ectoderm and in the presumptive neural retina.

Utilizing the 366 bp murine αA-crystallin promoter to drive CRE expression to the developing lens, *MLR10* and *MLR39* transgenes act later in development, and more lens-specifically, than those driven by the *Pax6* P0 promoter. Unlike the endogenous mouse αA-crystallin promoter, the 366 bp murine αA-crystallin promoter fragment lacks several lens-specific enhancer elements that support expression in the lens epithelium [26]. As a result, transgenes driven by this promoter fragment usually initiate expression around E12.5 and typically exclude the prenatal lens epithelium. The transgenic construct used to create the *MLR10* mice included a 20 bp consensus PAX6 binding site [27] embedded in the 366 bp mouse αA-crystallin promoter. CRE expression in *MLR10* mice initiates in the lens vesicle at E10.5 and mediates floxed DNA deletion in both the lens fiber cells and lens epithelium [8]. In addition, *MLR10* transgene expression in the eye remains lens-specific. In contrast, the *MLR39* transgene construct, consisting only of the 366 bp murine *αA-crystallin* promoter fragment, leads to the initiation of CRE expression in lens fiber cells at E12.5 without expression in the developing lens epithelium [8]. However, co-injection of the *αA-crystallin*/*CRE* construct with a mouse tyrosinase minigene produced light coat pigmentation in albino *MLR39* transgenic mice. Co-integration of the CRE transgene with the tyrosinase minigene, which normally leads to tyrosinase expression in melanocytes and retinal pigmented epithelium (RPE), provides an explanation of why this transgenic line initiates mosaic CRE expression in the RPE at E11.5.

A number of other CRE transgenes effectively delete floxed DNA in the developing lens, but these often exhibit a widespread deletion in other tissues. The *Nes-Cre* transgenic mice, for example, exhibit floxed gene deletion throughout the nervous system as well as in the lens epithelial cells and fiber cells [13, 14]. The *Ap2α-IRESCre* transgenic line catalyzes floxed DNA deletion in the presumptive lens ectoderm even earlier than those transgenic lines driven by the *Pax6* P0 promoter, but CRE expression in these mice includes large portions of head and trunk surface ectoderm and neural crest cells [28]. Also, the *Ap2α-IRESCre* mice resulted from targeted insertion of CRE into the endogenous *Tcfap2α* gene in embryonic stem cells rather than from zygote microinjection. While the *Nes-Cre* and *Ap2α-IRESCre* mice remain useful for lens gene deletion in particular contexts, the widespread, non-ocular expression exhibited by these transgenes limits their utility. For this reason, lens biologists more frequently use the more limited expression pattern of the *Le-Cre* and *MLR10* transgenic strains for gene deletion within the lens. Table 1 summarizes the published information currently available concerning the CRE transgenic lines with lens activity.

**Table 1:** Comparison of published CRE transgenic lines with lens expression

Name	Promoter elements	Method of creation	Lens expression	Non-lens expression	Status	Coding sequences	Notes
*Ap2α-IRESCre*	Endogenous mouse Tcfap2α gene	Embryonic stem cell targeted mutation	Head surface ectoderm prior to lens placode formation (before E9.0)	Extensive head and trunk surface ectoderm and neural crest cells	Available	CRE	IRES-CRE targeted to the 3’ UTR of the Tcfap2α gene
*Le-Cre*	6.5 Kb mouse Pax6 P0 promoter including both the ectodermal enhancer and retina/pancreas enhancer. Approximately 400 bp mouse Pax6 sequence follows the GFP coding sequence	Zygote microinjection	Lens placode and surrounding surface ectoderm by E9.5 with expression continuing in lens epithelium	Endocrine pancreas, all surface ectoderm-derived eye structures, epidermis from eye to snout	Available	CRE and GFP	Nuclear-localized CRE separated from GFP by an IRES. Microphthalmia in homozygotes and genetic background-dependent variable ocular abnormalities in hemizygotes
*LR-Cre*	Three tandem copies of the mouse Pax6 P0 ectodermal enhancer upstream of the minimal P0 promoter	Zygote microinjection	Lens placode and surrounding surface ectoderm by E9.5	Optic vesicle by E9.5	Available	CRE and GFP	CRE separated from GFP by an IRES
*MLR10*	366 bp mouse αA-crystallin promoter with an internal insertion of a 20 bp Pax6 consensus binding site	Zygote microinjection	Lens vesicle by E10.5–11	Snout and vibrissae follicles, as well as in parts of the midbrain and pituitary gland by E12.5	Available	CRE	
*MLR39*	366 bp mouse αA-crystallin promoter driving CRE with co-injected Tyrosinase minigene	Zygote microinjection	Lens fiber cells by E12.5	Mosaic RPE expression of CRE	Available	CRE and tyrosinase	Co-injection of both the CRE and tyrosinase transgene resulted in light coat and RPE pigmentation. CRE restricted to lens fibers and RPE
*mαA-Cre*	366 bp mouse αA-crystallin promoter	Zygote microinjection	Assumed in lens fiber cells by E12.5	Unknown	Extinct	CRE	First demonstrated use of CRE recombination in transgenic mice
*Nes-Cre*	Approximately 5 kb rat nestin promoter with nestin nervous system-specific enhancer following the human growth hormone poly adenylation signal	Zygote microinjection	Lens epithelium and fiber cells by E14.5	Central and peripheral nervous system, ciliary body and isolated expression in the heart and kidney	Available	CRE	Widespread non-lens expression including retina and ciliary body within the eye
*P0-3.9GFPCre*	3.9 Kb mouse Pax6 P0 promoter including both the ectodermal enhancer and retina/pancreas enhancer	Zygote microinjection	Lens placode and surrounding surface ectoderm by E9.5 with expression continuing in lens epithelium	Endocrine pancreas, stomach mesenchyme, apical ectodermal ridge, all surface ectoderm-derived eye structures, epidermis from eye to snout	Available	CRE and GFP	CRE and GFP are expressed as a fusion gene
*Pax6(lens)-Cre*	Minimal mouse P0 Pax6 promoter preceded by a single copy of the 340 bp ectodermal enhancer	Zygote microinjection	Lens placode by E9.5	Unknown	Extinct	CRE	

Despite its widespread use, the *Le-Cre* transgene sometimes disrupts lens development independent of any floxed DNA deletion. The first report of cataracts and microphthalmia associated with homozygous *Le-Cre* transgenic mice appeared more than a decade ago [29]. More recently, investigators have documented lens and cornea abnormalities in *Le-Cre* hemizygous mice, the severity of which varied on different genetic backgrounds [30, 31]. These observations demonstrate the necessity of including hemizygous *Le-Cre-*only control mice in addition to animals homozygous for the floxed allele but lacking the *Le-Cre* transgene. The independent ocular phenotypes resulting from the *Le-Cre* transgene raise concerns about these previous studies that failed to include *Le-Cre* controls. *FVB/N* inbred mice provided the original genetic background for the *Le-Cre* transgene, and hemizygous *Le-Cre* transgenics on this background typically appear identical to non-transgenic *FVB/N* mice. However, the *Le-Cre* transgene, even in hemizygous state, may sensitize the lens in such a way as to exacerbate the phenotype resulting from simple loss of the floxed DNA segment under investigation. In this case, the *Le-Cre* transgene may actively enhance the phenotype rather than solely mediating the homozygous mutation. These concerns motivate the search for an alternative transgene to mediate gene deletions in the early developing lens or, at minimum, indicate the need for additional controls in the case of *Le-Cre*.

Given the available CRE transgenic lines, the *P0-3.9GFPCre* transgenic mice represent the most likely replacement for the *Le-Cre* mice, since both of these lines utilize the *Pax6* P0 promoter that leads to CRE expression in the lens placode. The *P0-3.9GFPCre* mice have no reported ocular abnormalities in hemizygous or homozygous state. However, few studies have utilized the *P0-3.9GFPCre* transgenic mouse line and no comprehensive analysis of its tissue-specific CRE expression pattern exists. Here, we compare expression data for *Le-Cre* and *P0-3.9GFPCre* mice. Although the *P0-3.9GFPCre* lack the ocular defects sometimes seen in *Le-Cre* mice, we show widespread CRE expression in *P0-3.9GFPCre* mice prior to E8.0 that leads to floxed gene deletion in multiple, non-ocular tissues.

## Results

**Fig. 2:**
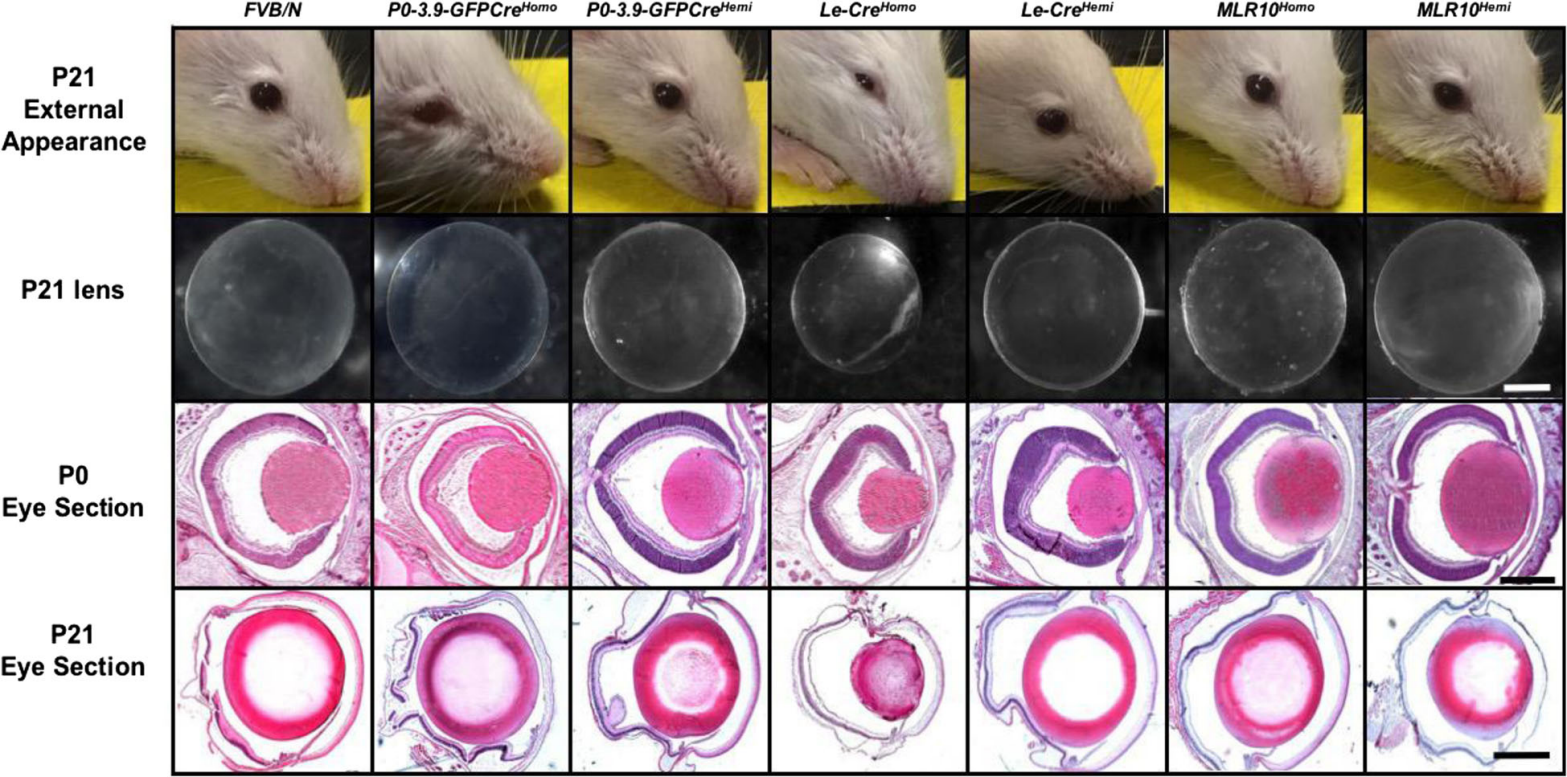
Gross comparison of ocular development in wild-type *FVB/N* mice and *P0-3.9GFPCre*, *Le-Cre*, and *MLR10* transgenic mice. All genotypes exhibit grossly normal appearing eyes and lenses at all stages examined, with the exception of mice homozygous for the *Le-Cre* transgene (*Le-Cre*^*Homo*^). Homozygous *Le-Cre* mice (middle column) exhibit externally obvious microphthalmia and small lenses at 3 weeks after birth (P21). Homozygous *Le-Cre* lenses exhibit consistent size reduction with evidence of fiber cell disorganization and nuclear retention that progressively worsens with age. As all mice were maintained on an *FVB/N* inbred background, all P21 eye sections exhibit photoreceptor degeneration from rd1 homozygous mutation [57]

In contrast to *Le-Cre* mice, no obvious ocular abnormalities appear in homozygous *P0-3.9GFPCre* mice. Both the *Le-Cre* mice and *P0-3.9GFPCre* mice originated on an *FVB/N* genetic background, and neither of these transgenic lines produce animals with conspicuous ocular abnormalities on this genetic background in hemizygous state. However, while the eyes from homozygous *P0-3.9GFPCre* mice appear normal, *Le-Cre* homozygous transgenic mice consistently exhibit smaller eyes and lenses with varying degrees of lens size reduction, fiber cell disorganization, and cataracts on all inbred and mixed genetic backgrounds tested, including *FVB/N* (Fig. 2).

Real-time transgene expression and lineage tracing data provide different views of CRE expression during development. Since both the *Le-Cre* and *P0-3.9GFPCre* transgenes co-express CRE and GFP (green fluorescent protein), green fluorescence provides an excellent, easily visualized way to compare the real-time expression pattern of these transgenes during embryonic development. Nevertheless, real-time analysis fails to reveal CRE expression that may have occurred at an earlier developmental time point. A number of CRE-reporter mice exist that provide information about the cellular descendants of cells in which CRE expression occurs at any time during development. In particular, the original *Gt (ROSA) 26Sor*^*tm1Sor*^ (*R26R*) transgenic mice possess a ubiquitously active promoter separated from a *lacZ* gene by a floxed *pgk-neo-polyA* antibiotic resistance cassette [32]. CRE-mediated deletion of the neo cassette results in *lacZ* expression, visualized by X-Gal histochemistry, in all cellular descendants, irrespective of whether CRE expression persists.

**Fig. 3:**
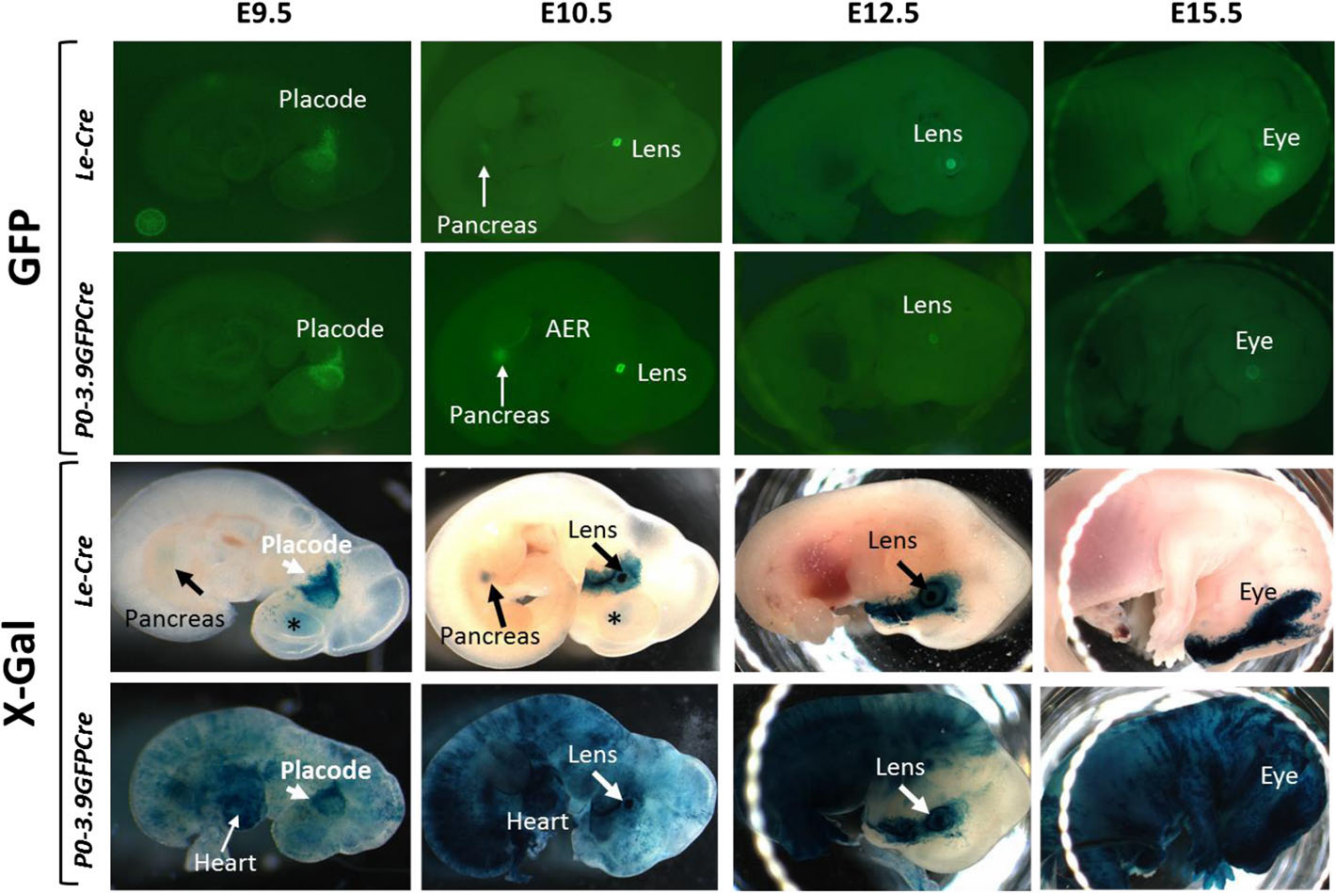
Comparison of *Le-Cre* and *P0-3.9GFPCre* embryos using both real-time transgene expression and lineage tracing. GFP (top two rows) co-expressed with the CRE transgene in both *Le-Cre* and *P0-3.9GFPCre* mice made it possible to compare real-time transgene expression of both transgenes in whole mount embryos. X-Gal staining (bottom two rows) in embryos carrying both the ROSA26 CRE reporter (*Gt(ROSA)26Sor*^*tm1Sor*^) and either the *Le-Cre* or *P0–3.9GFPCre* transgene provided an alternate way to compare the CRE transgenic lines based on lineage tracing of CRE expressing cells. *Le-Cre* (first row) and *P0-3.9GFPCre* (second row) transgenic embryos exhibit largely identical GFP expression patterns from E9.5 through E15.5 with the exception of expression in the apical ectodermal ridge (AER) of the forelimb observed specifically in the *P0-3.9GFPCre* transgenic embryos at E10.5. In contrast to the largely identical pattern of GFP expression, X-Gal staining revealed marked differences in expression between the *Le-Cre* (third row) and *P0-3.9GFPCre* (fourth row) transgenic embryos. Although *P0-3.9GFPCre* exhibited extensive embryo-to-embryo variability, all embryos from this strain exhibited extensive non-ocular X-Gal staining patterns at every stage examined. Externally visible X-Gal staining in the *Le-Cre* embryos remained restricted to the eye and surface ectoderm surrounding the eye proceeding in a streak of ectoderm toward the developing snout and the pancreas. Some X-Gal staining in the forebrain showed through the surface ectoderm at E9.5 and E10.5 in *Le-Cre* embryos (asterisks)

Real-time analysis, based on GFP fluorescence, revealed similar patterns of transgene expression in *Le-Cre* and *P0-3.9GFPCre* transgenic embryos at E9.5, E10.5, E12.5, and E15.5 (Fig. 3). At E9.5, both lines exhibited obvious GFP expression in the head surface ectoderm surrounding and including the lens placode. GFP expression in both lines became more restricted to the lens and pancreas at E10.5, while a thin line of GFP expression in the apical ectodermal ridge of the forelimb appeared only in the *P0-3.9GFPCre* transgenic embryos. E12.5 and E15.5 embryos expressed externally visible GFP only in the eye with fluorescence intensity appearing stronger in the *Le-Cre* embryos than in the *P0-3.9GFPCre* embryos.

Despite the similar GFP expression patterns seen in both lines, lineage-tracing analysis, based on LacZ expression (visualized by X-Gal), revealed distinct differences between *Le-Cre* and *P0-3.9GFPCre* transgenic embryos. Blue X-Gal staining in the E9.5 *Le-Cre* embryos appeared in a bilateral swath of head surface ectoderm encompassing the lens placode, matching the GFP expression pattern at the same stage. However, X-Gal staining appeared widespread in the E9.5 *P0-3.9GFPCre* embryos, with abundant expression in both the lens placode and the heart (Fig. 3).

Lineage tracing revealed divergent expression patterns of CRE-mediated gene deletion between *Le-Cre* and *P0-3.9GFPCre* embryos subsequent to lens formation. As development progressed beyond E9.5, *Le-Cre* lenses exhibited deep blue staining with the surrounding surface ectoderm appearing more lightly stained and forming a blue streak encompassing the eye and extending to the end of the snout (Fig. 3, X-Gal, *Le-Cre* E10.5–E15.5). By E15.5, closure of the developing eyelid obscured the blue staining within the lens. Elsewhere, X-Gal staining in the developing pancreas appeared through the translucent *Le-Cre* embryos at E9.5 and E10.5. Likewise, many *Le-Cre* embryos exhibited some X-Gal staining in the developing forebrain at E9.5–E10.5 (Fig. 3, asterisks). As the overlying tissues thickened in the *Le-Cre* embryos, the internal X-Gal staining in the pancreas and forebrain faded from view (Fig. 3, X-Gal, *Le-Cre* E12.5, E15.5). Although *P0-3.9GFPCre* embryos exhibited considerable embryo-to-embryo variability, most E10.5–E15.5 embryos exhibited extensive, mosaic X-Gal staining throughout the entire embryo surface (Fig. 3, bottom row).

**Fig. 4:**
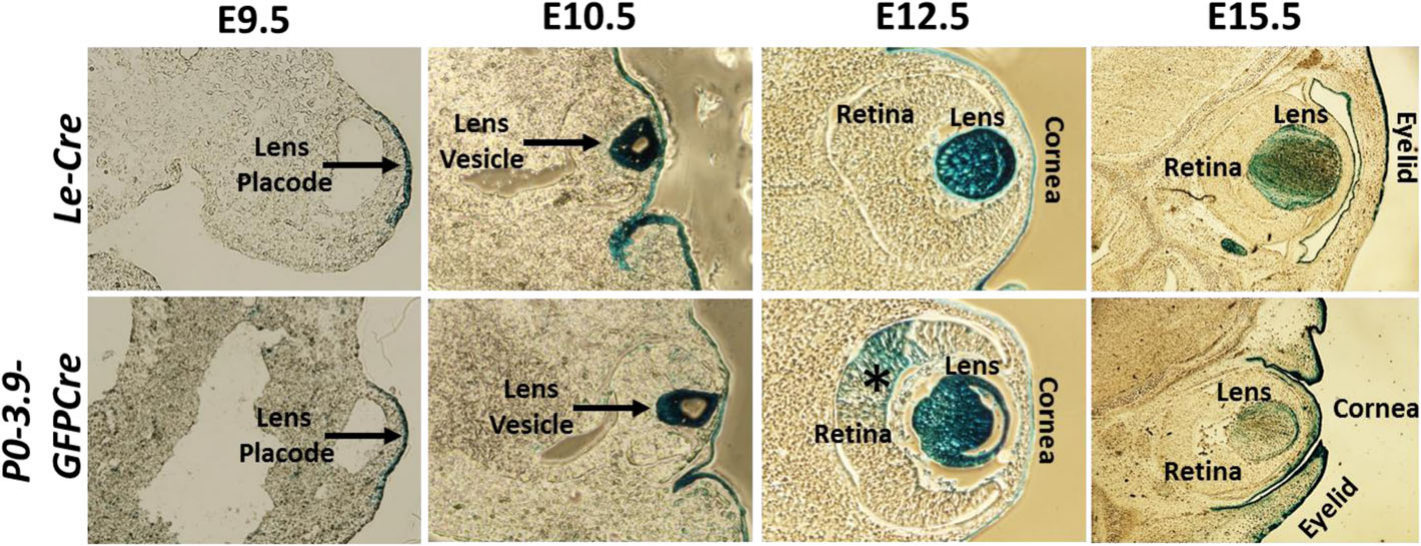
Comparison of X-Gal staining patterns in *Le-Cre* and *P0-3.9GFPCre* eye sections. Although the ocular pattern of blue X-Gal staining appeared similar in *Le-Cre* (top row) and *P0-3.9GFPCre* (bottom row) embryos, patchy retinal X-Gal staining only appeared in *P0-3.9GFPCre* embryos (asterisk at E12.5)

Sections through the developing eye localized the X-Gal staining in the *Le-Cre* embryos exclusively in the lens placode at E9.5 and in the developing lens and ocular surface ectoderm derivatives (corneal epithelium, developing conjunctiva, and eyelid surface) from E10.5–E15.5 (Fig. 4). Sections through *P0-3.9GFPCre* embryo heads revealed a similar pattern of X-Gal staining at E9.5 and E10.5. Alternatively, *P0-3.9GFPCre* eyes often exhibited patchy X-Gal staining in the retina (see Fig. 4, E12.5, asterisk) and more extensive surface ectoderm staining than seen in the *Le-Cre* embryos (see Fig. 4, E15.5).

**Fig. 5:**
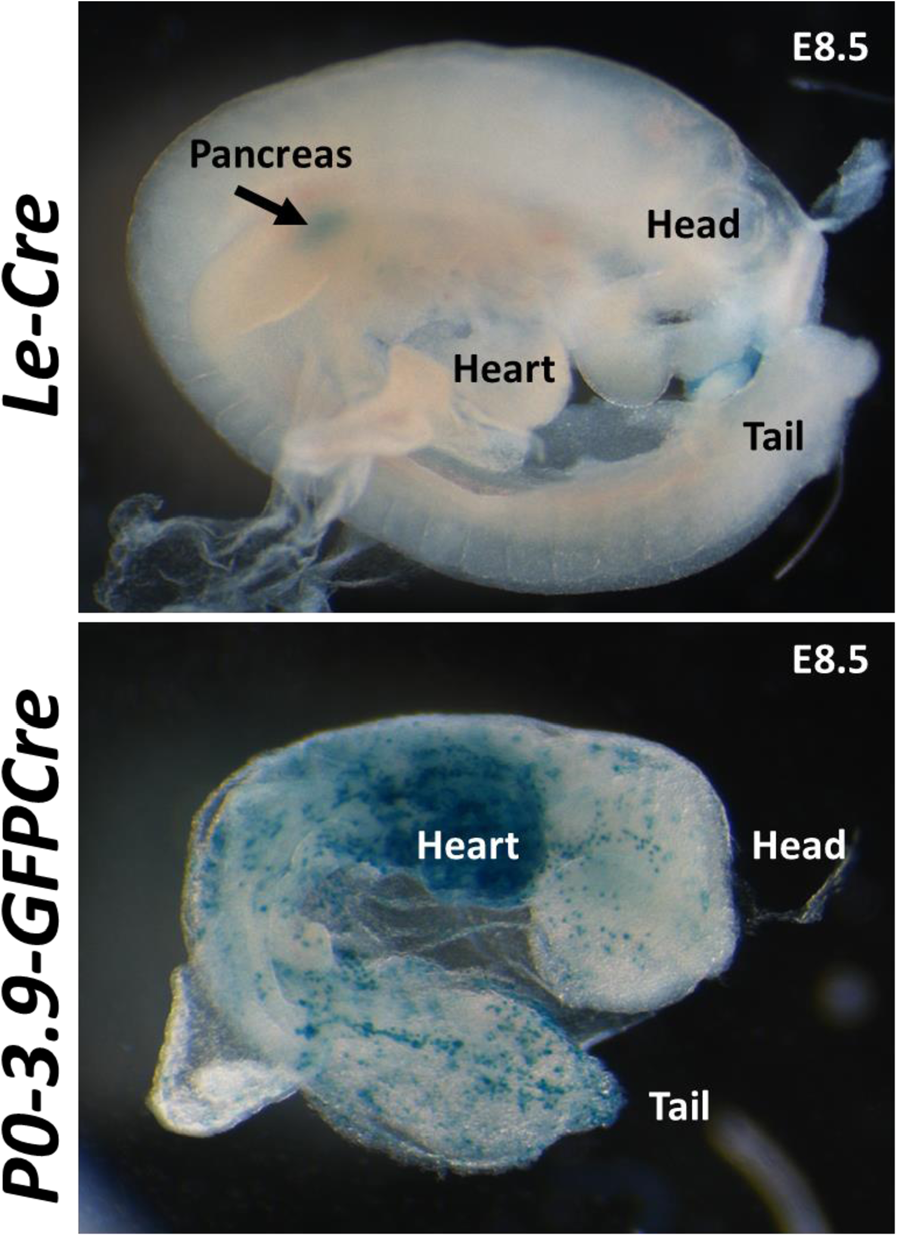
*P0-3.9GFPCre* E8.5 embryos exhibit extensive CRE-mediated recombination prior to lens placode formation. At E8.5, X-Gal staining in *Le-Cre* embryos (top) remained restricted to the developing pancreas. In contrast, *P0-3.9GFPCre* embryos (bottom) exhibited many patches of X-Gal stained tissue throughout the embryo, with particularly high numbers of blue clones in the developing heart region

To investigate the origin of the divergent X-Gal staining pattern observed between *Le-Cre* and *P0-3.9GFPCre* embryos, we examined embryos from each strain at E8.5 (Fig. 5). At this stage, weak X-Gal staining in *Le-Cre* embryos appeared only in the developing pancreas. In contrast, the E8.5 *P0-3.9GFPCre* embryos exhibited an extensive speckled pattern of X-Gal staining throughout much of the embryo with a relatively greater concentration of stained cells visible in the area of the developing heart. This early mosaic pattern of CRE expression in *P0-3.9GFPCre* embryos, prior to E9.0, is consistent with the extensive non-ocular X-Gal staining pattern seen in older embryos.

**Fig. 6:**
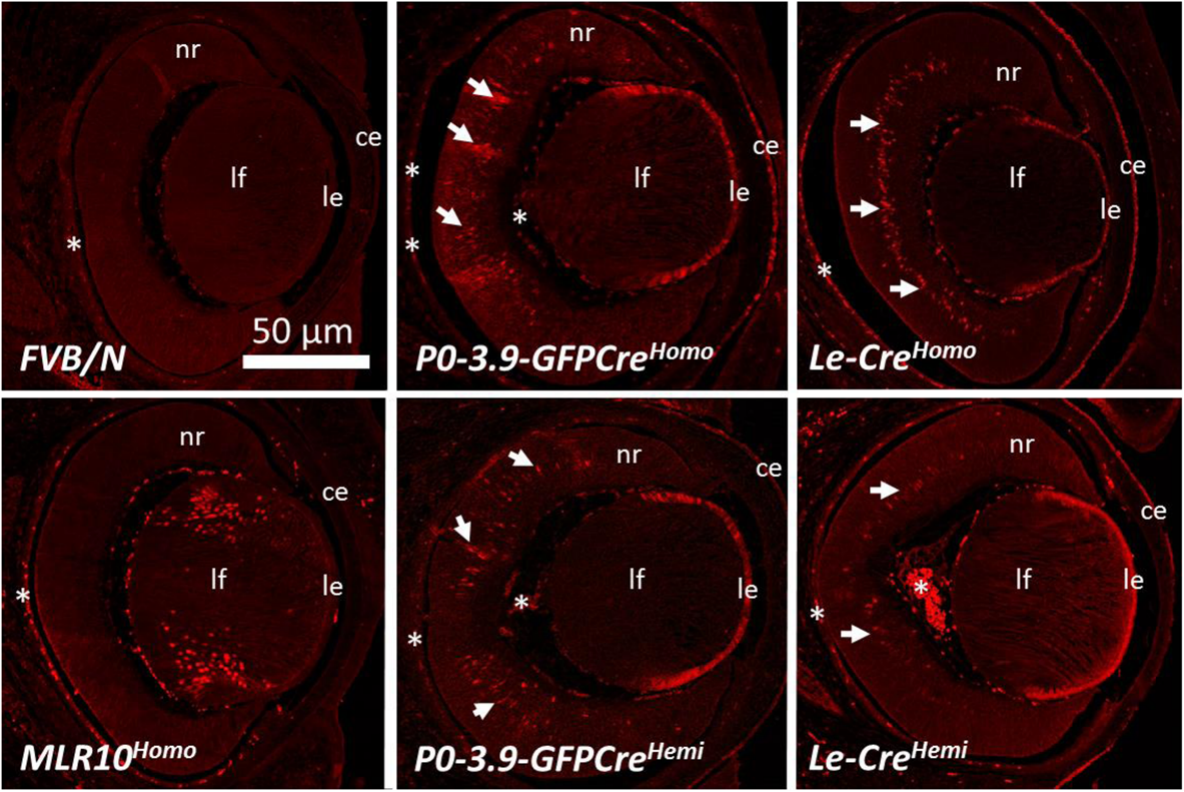
Immunohistochemical detection of CRE protein in *P0-3.9GFPCre*, *Le-Cre*, and *MLR10* transgenic mice at E15.5. *MLR10* exhibited CRE protein expression specifically in the nuclei of differentiating lens fiber cells (lf) within the eye at E15.5, while *P0-3.9GFPCre*, *Le-Cre* lenses showed obvious CRE protein in the epithelium of both the lens (le) and cornea (ce) and numerous cells within the developing neural retina (nr) indicated by arrows. Notice that the homozygous *Le-Cre* (*Le-Cre*^*Homo*^) lens is specifically small and misshapen relative to the lenses from the other genotypes. The *FVB/N* lens exhibits no specific staining with the anti-CRE antibody and serves as a negative control. Autofluorescence in blood cells in the choroid and *tunica vasculosa lentis* represent non-specific signal (asterisks). Transgenic homozygotes and hemizygotes are indicated by homo and hemi, respectively

Immunohistochemistry, using an antibody to CRE, provided a more direct assessment of CRE expression in the eyes of *Le-Cre* and *P0-3.9GFPCre* embryos (Fig. 6). Comparisons were made between hemizygous and homozygous E15.5 eyes from both *Le-Cre* and *P0-3.9GFPCre* embryos with non-transgenic *FVB/N* and homozygous *MLR10* transgenic eyes providing negative and positive controls for CRE expression, respectively. Homozygous *Le-Cre* mice uniquely exhibit reduced lens size, despite both *Le-Cre* and *P0-3.9GFPCre* mice exhibiting a similar CRE expression pattern in both the lens epithelium and in the corneal epithelium. CRE expression appeared in the developing neural retina in both *Le-Cre* and *P0-3.9GFPCre* eyes (Fig. 6, arrows). The CRE positive nuclei in the *Le-Cre* retina formed a reasonably tight layer in the middle of the posterior half of the retina. In the *P0-3.9GFPCre* retina, CRE positive nuclei appeared more dispersed throughout the thickness of the posterior half of the retina. In both strains, CRE expression in the retina appeared stronger in the homozygous transgenic embryos. These cells are likely postmitotic because they appear restricted to the developing inner nuclear layer. For comparison, *MLR10* mice exhibit normal ocular size with CRE protein expression most prominent in the elongating lens fiber cells at E15.5, with no ocular CRE expression outside the lens.

Although the extra-ocular CRE expression pattern of *Le-Cre* and *P0-3.9GFPCre* mice differed substantially, the patterns within the lens appeared similar. At E15.5, the lens expression pattern of *MLR10* mice appeared largely reciprocal to that of the other two CRE transgenic strains. *Le-Cre* and *P0-3.9GFPCre* expression appeared in the epithelium with no evidence of fiber cell CRE expression while the CRE protein in the *MLR10* mice appeared exclusively in the nuclei of differentiating fiber cells. Despite the lack of CRE protein in the *MLR10* E15.5 lens epithelium, *MLR10* mice do express CRE in the lens epithelial precursors at an earlier stage of development [8].

**Fig. 7:**
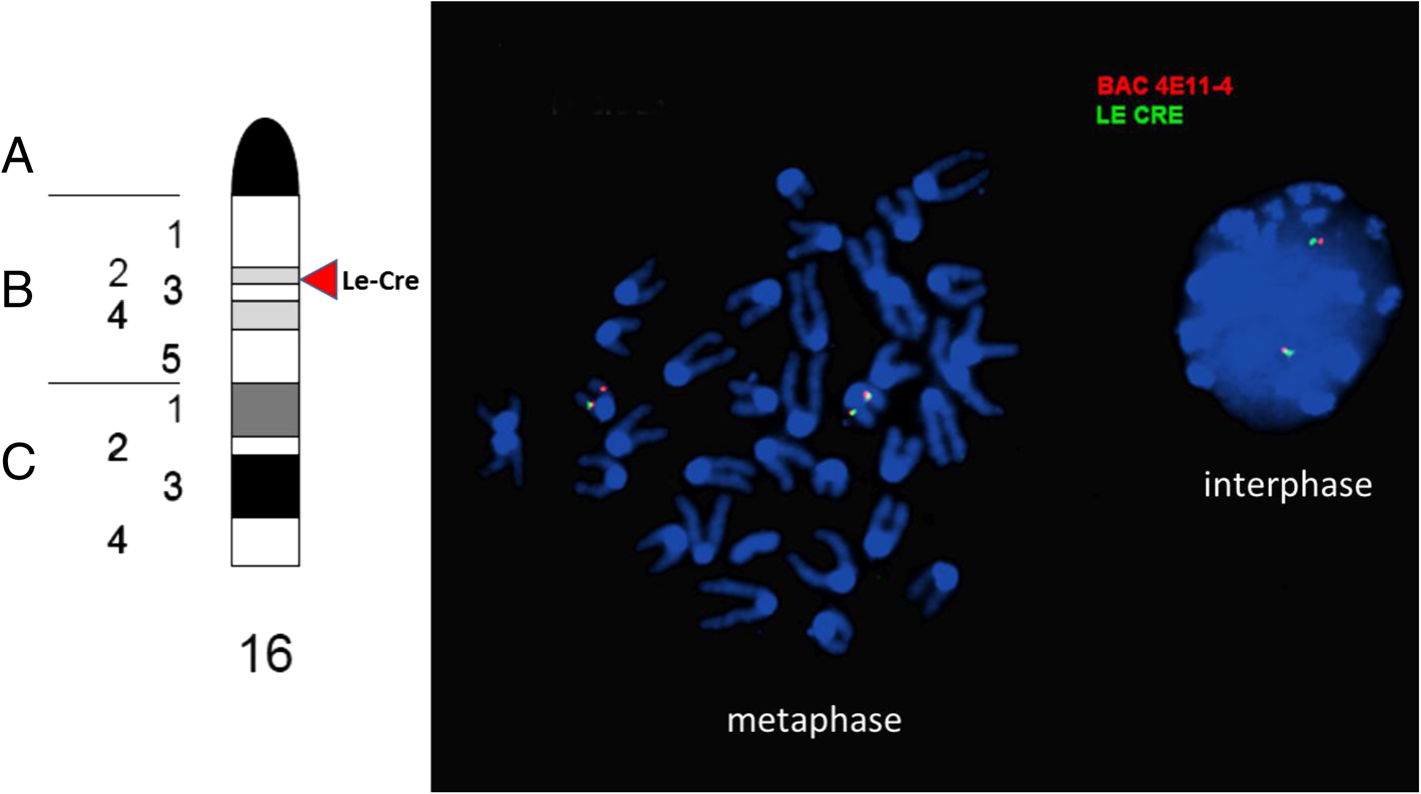
Fluorescence in situ hybridization (FISH) localization of the *Le-Cre* transgene to chromosome 16. Co-localization of fluorescently labeled DNA probes for both CRE (green signal) and BAC DN-4E11, a mouse BAC from chromosome 16 (red signal) confirmed the chromosomal location of the *Le-Cre* transgene insertion. The approximate cytogenetic location of the Le-Cre transgene is indicated by a red arrowhead on the mouse chromosome 16 idiogram (from the Idiogram Album by David Adler© 1994) on the left side of the figure

Given the similar lens expression pattern of the *Le-Cre* and *P0-3.9GFPCre* mice, we conducted further analyses in an attempt to explain why ocular abnormalities occur in all *Le-Cre* homozygotes and some *Le-Cre* hemizygotes [30]. The microphthalmia seen in homozygous *Le-Cre* mice could arise via insertional mutation if transgene sequences disrupt gene coding sequences or cis-regulatory elements. We used fluorescence in situ hybridization (FISH) to localize the *Le-Cre* transgene sequences to mouse chromosome 16, nearby and telomeric to a BAC clone DN-4E11 (Fig. 7). The *Le-Cre* strain in use today represents one of two founder lines produced via microinjection of the identical construct into *FVB/N* zygotes [9]. Homozygous transgenic mice derived from a second founder also developed microphthalmia (Dr. Ruth Ashery-Padan-unpublished data). The line established from the second founder (RD6) is extinct, but fibroblasts established from the last surviving mouse from this line were used for FISH to establish that the insertion site for this transgene was on chromosome 10, based on co-hybridization with BAC clone RP24-257H11 (Additional file 1: Figure S1). The similar homozygous phenotype of two independent transgenic lines derived by inserting the same microinjection construct into two different loci makes it unlikely that microphthalmia in both *Le-Cre* transgenic lines results from independent insertional mutations.

**Fig. 8:**
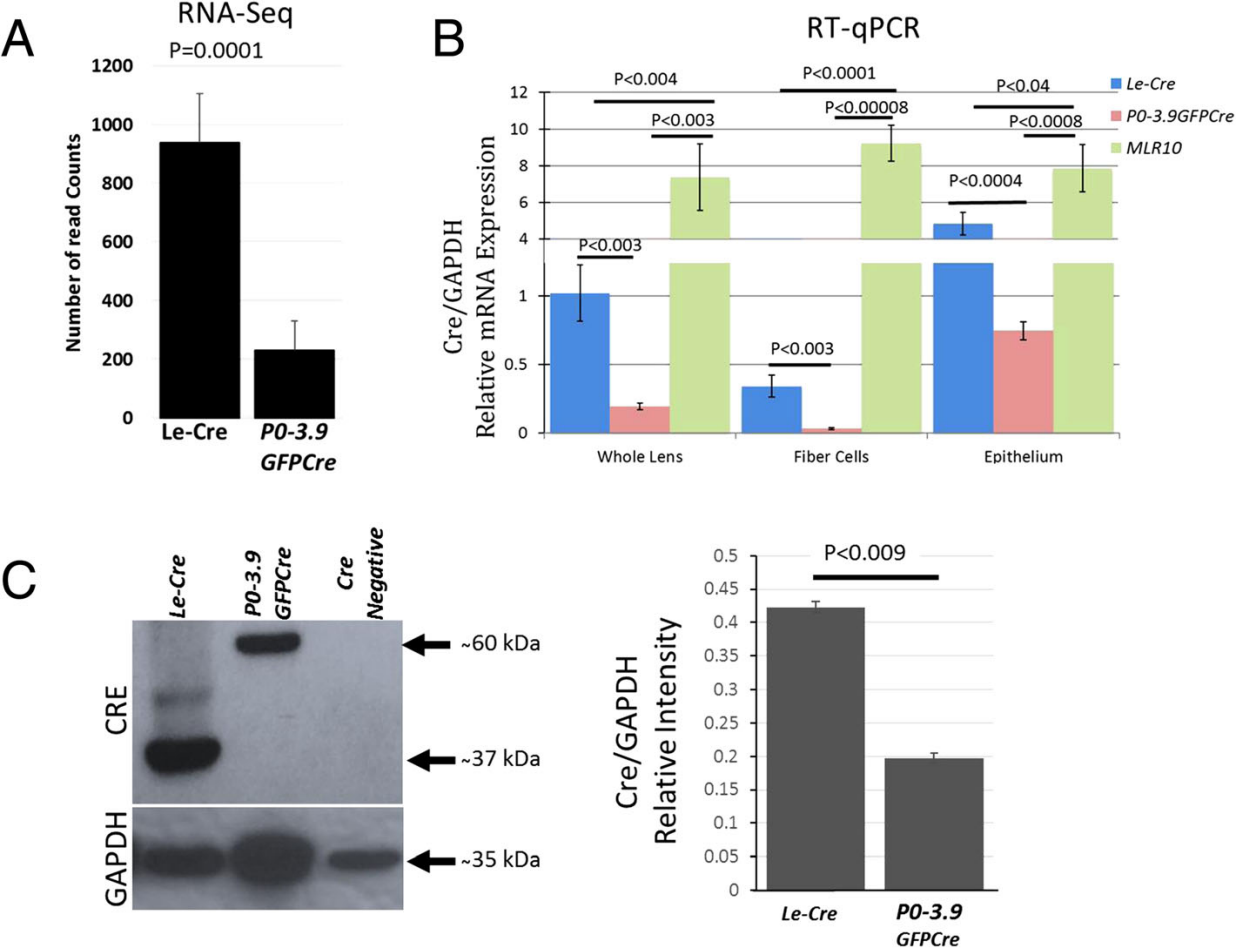
Cre expression in *Le-Cre* and *P0-3.9GFPCre* lenses. RNA-Seq analysis detected significantly more CRE reads in newborn lens RNA from the *Le-Cre* mice than from *P0-3.9GFPCre* mice (**a**). Quantitative RT-PCR analysis confirmed the increase in CRE mRNA in newborn *Le-Cre* lenses relative to *P0-3.9GFPCre* lenses (**b**). Western blots revealed that newborn *Le-Cre* lenses also expressed relatively more CRE protein than *P0-3.9GFPCre* lenses (**c**, blot). Image-J analysis showed that, relative to GAPDH, the *Le-Cre* lenses expressed nearly twice as much CRE protein as the *P0-3.9GFPCre* lenses (**c**, graph). Error bars indicate standard error of the mean calculated by a two-tailed Student’s *t* test. Each bar in this figure represents the average of three biological replicates and three technical replicates

Because overexpression of CRE can induce toxicity in some cell types [33–37], we quantitatively examined CRE RNA expression in newborn hemizygous *Le-Cre* and *P0-3.9GFPCre* lenses. RNA-Seq analysis of whole newborn lenses showed a significantly higher expression (approximately fourfold normalized reads) of CRE transcripts in the *Le-Cre* lenses than the *P0-3.9GFPCre* lenses (Fig. 8a). To explore this result further, we measured CRE transcripts in newborn lenses from hemizygous *Le-Cre*, *P0-3.9GFPCre*, and *MLR10* mice by RT-qPCR. In addition, we analyzed RNA from dissected lens fiber cells and cells adherent to the lens capsule (enriched for lens epithelial cells). The *MLR10* lens RNA samples contained approximately 7-fold and 35-fold more CRE transcripts than whole lens RNA samples from *Le-Cre* and *P0-3.9GFPCre* lenses, respectively (Fig. 8b). As expected, both the *Le-Cre* and *P0-3.9GFPCre* lenses contained more CRE transcripts in the epithelial cell RNA fraction than in the fiber cell RNA fraction. Despite this, the RT-qPCR analysis revealed no significant difference between CRE transcript abundance derived from fiber cell and epithelial cell RNA fractions from *MLR10* lenses.

To determine if the larger number of CRE transcripts in the *Le-Cre* lenses relative to the *P0-3.9GFPCre* lenses correlated with an increased abundance of CRE protein, we performed western blot analysis on newborn lenses using a specific CRE antibody (Fig. 8c). As expected, the CRE immunoreactive band from the *Le-Cre* lenses appeared lower (37 kDa) than the band from GFP-CRE fusion protein expressed by the *P0-3.9GFPCre* lenses (60 kDa). Quantitative analysis of the relative signal intensity of the CRE immunoreactive band to the GAPDH immunoreactive loading control revealed that newborn *Le-Cre* lenses expressed approximately twice the amount of CRE protein than newborn *P0-3.9GFPCre* lenses (Fig. 8c). No CRE immunoreactivity was observed in *FVB/N* lens protein.

**Fig. 9:**
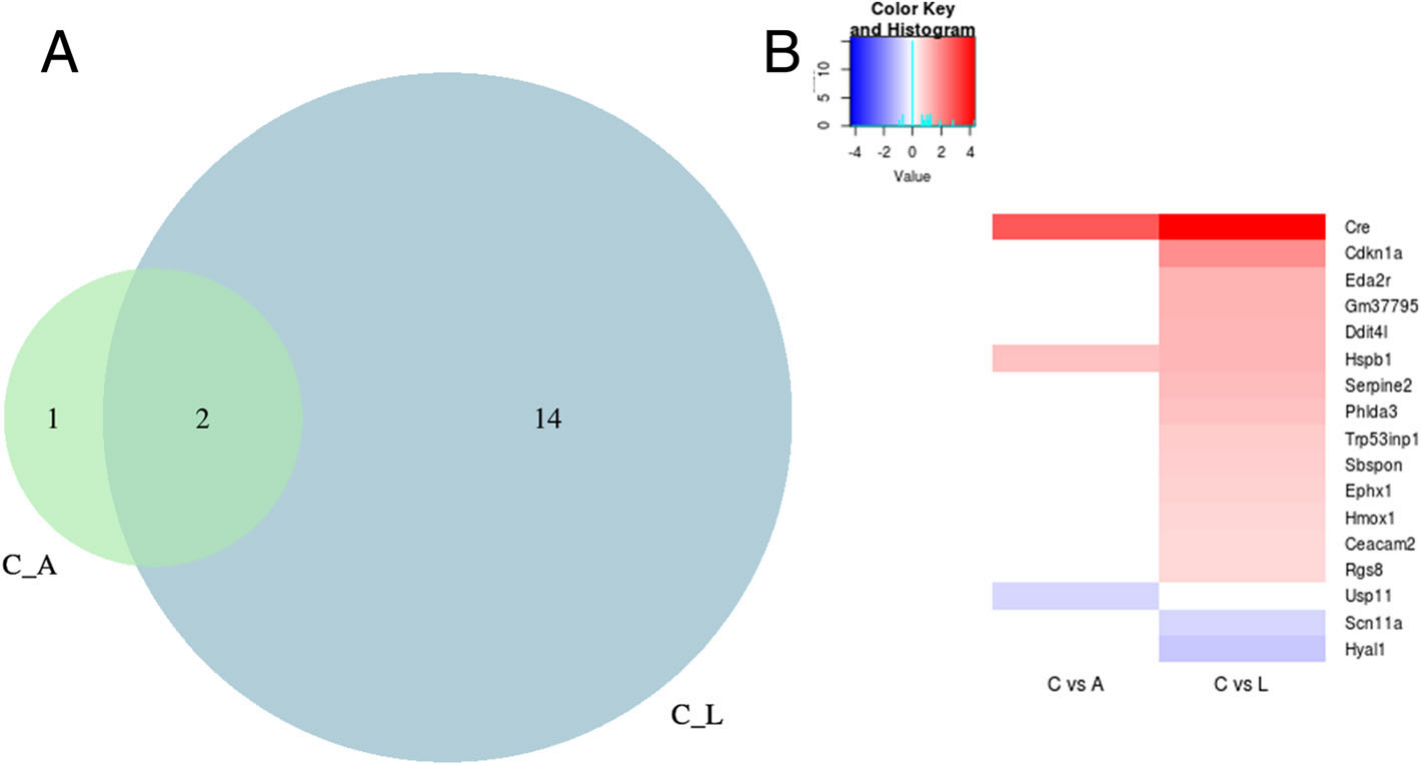
Differential gene expression in hemizygous newborn *Le-Cre* and *P0-3.9GFPCre* lenses. The expression of transcripts expressed in newborn *Le-Cre* and *P0-3.9GFPCre* lenses were compared to newborn lens transcripts from the background *FVB/N* strain mice by RNA-Seq analysis. A Venn diagram (**a**) illustrates the total number of genes differentially expressed by the transgenic lenses versus the *FVB/N* lenses while the heatmap (**b**) lists all of the transcripts differentially expressed with red indicating overexpression and blue indicating underexpression. The *Le-Cre* lenses differentially expressed 16 genes while the *P0-3.9GFPCre* lenses differentially expressed only three genes. Both transgenic lenses overexpressed CRE and Hspb1 transcripts. The *P0-3.9GFPCre* lenses uniquely underexpressed Usp11. Many of the overexpressed transcripts in the *Le-Cre* lenses (Cdkn1a, Hmox1, Phlda3, and Trp53inp1) are associated with apoptosis and/or stress response (Hspb1, Ephx1)

Given the phenotypic differences between *Le-Cre* and *P0-3.9GFPCre* homozygous lenses, we sought to determine if other genes were differentially expressed in hemizygous lenses from these strains, relative to non-transgenic *FVB/N* lenses. Relative to the *FVB/N* background strain, hemizygous *P0-3.9GFPCre* lenses only showed significant upregulation of two genes, including CRE and downregulation of one gene (Fig. 9). In contrast, the hemizygous *Le-Cre* lenses significantly upregulated 14 genes and downregulated two genes. Although neither CRE nor GFP is expressed in *FVB/N* lenses, we specifically measured CRE expression relative to GAPDH in all three lines but did not measure GFP expression.

Although neither transgenic line differentially expressed many genes in the hemizygous lens, the Le-Cre lenses differentially expressed more than five times the number of genes than seen in the *P0-3.9GFPCre* lenses. Transgenic lenses from both lines overexpressed *Hspb1* transcripts relative to *FVB/N* lenses. *Hspb1* encodes a small heat shock protein (HSP25 in mouse/HSP27 in humans) [38] that acts as stress-induced chaperone involved in the regulation of cell cycle arrest, cell differentiation, and apoptosis [39]. RNA-Seq analysis from newborn lens RNA revealed that the *Le-Cre* lenses specifically upregulated several genes associated with apoptosis or cell growth. These include *Cdkn1a*, *Hmox1*, *Phlda3*, *Serpine2*, and *Trp53inp1*. Of these deregulated transcripts, Cdkn1a exhibits the highest overexpression. The *Cdkn1a* gene encodes the cyclin-dependent kinase inhibitor, p21^Cip1^, a potent cell cycle inhibitor and target for p53-mediated apoptosis [40]. Downregulated genes included *Usp11* for *P0-3.9GFPCre* and *Scn11a* and *Hyal1* for *Le-Cre*. USP11 acts as a deubiquitylase that can stabilize a number of protein substrates including p53 [41] and p21^CIP1^ [42]. *Scn11A* encodes a voltage-gated sodium channel protein (Nav1.9) that is preferentially expressed in sensory neurons and associated with pain perception [43]. *Hyal1* encodes a hyaluronidase implicated in extracellular matrix degradation and epithelial-mesenchymal transition [44]. Notably, none of the genes encoding important transcription factors involved in lens development (*Pax6*, *Six3*, *c-Maf*, *Pitx3*, *Hes1*, *Prox1*, *Hsf4*, *Sox2*, *Sox1,* etc.) [45] are deregulated in the lenses of either transgenic line. Significant gene ontology (GO) terms for the differentially expressed genes in the *Le-Cre* and *P0-3.9GFPCre* lenses are listed in Table 2.

**Table 2:** Gene ontology terms associated with differentially regulated genes in Le-Cre lenses

GO term	Count	*p* value	Genes
Negative regulation of cell proliferation	4	3.6 × 10^−3^	*Cdkn1a*, *Hmox1*, *Serpine2*, *Trp53inp1*
Negative regulation of cell growth	3	4.7 × 10^−3^	*Cdkn1a*, *Hyal1*, *Serpine2*
Cellular response to UV-B	2	6.6 × 10^−3^	*Cdkn1a*, *Hyal1*
Intrinsic apoptotic signaling pathway in response to DNA damage by p53 class mediator	2	2.5 × 10^− 2^	*Cdkn1a*, *Phlda3*
Positive regulation of apoptotic process	3	3.1 × 10^−2^	*Hmox1*, *Phlda3*, *Trp53inp1*
Cellular response to heat	2	3.2 × 10^−2^	*Cdkn1a*, *Hmox1*
Negative regulation of signal transduction	2	4.3 × 10^−2^	*Ddit4l*, *Rgs8*
Cell cycle arrest	2	6.5 × 10^−2^	*Cdkn1a*, *Trp53inp1*
Response to toxic substance	2	6.9 × 10^−2^	*Cdkn1a*, *Ephx1*
Apoptotic process	3	7.9 × 10^−2^	*Hmox1*, *Phlda3*, *Trp53inp1*
Positive regulation of angiogenesis	2	9.6 × 10^−2^	*Hmox1*, *Hyal1*

## Discussion

Cre recombinase provides an important tool for genome manipulation. This remains true in studies of lens development and physiology, the system that first demonstrated the utility of CRE in transgenic mice. Precisely, how to deliver CRE to the lens is a relevant issue in any experimental context. The currently available transgenic strains each have advantages and disadvantages that merit consideration prior to conducting experiments in living animals.

Of the transgenic lines that express CRE in the lens, the *Le-Cre* and *MLR10* lines have proven most popular. Lenses from homozygous *MLR10* mice appear normal, suggesting a lack of CRE-toxicity. Ocular CRE expression within *MLR10* mice only occurs in the lens, but this line does not express CRE in the lens placode and may exhibit incomplete recombination in the lens epithelium with some floxed alleles. Alternatively, the *Le-Cre* transgenic mice exhibit excellent recombination of floxed alleles in the lens placode resulting in floxed allele deletion in the lens, corneal epithelium, conjunctiva, lacrimal glands, and eyelids. Nevertheless, CRE expression in *Le-Cre* mice also recombines floxed alleles in the endocrine pancreas. Our detection of CRE immunoreactivity in the developing retina of both *Le-Cre* and *P0-3.9GFPCre* mice (Fig. 6) suggests that floxed allele deletion occurs in a subset of the retina in these mice as well. Curiously, at E15.5, we did not detect CRE-mediated recombination in the *Le-Cre* retina using CRE reporter transgenes (Fig. 4). This might represent a delay between the onset of retinal CRE expression and having enough Lac Z protein expressed to show up in the X-Gal reporter assay. The CRE expressing cells in the retina, based on their number and location (Fig. 6), are likely precursors of a subset of amacrine interneurons and horizontal cell precursors, previously identified in fate mapping experiments using a 450 bp enhancer fragment included in both the *Le-Cre* and *P0-3.9GFPCre* constructs [23].

The consistent ocular abnormalities seen in *Le-Cre* homozygotes coupled with the genetic background-dependent ocular phenotypes in *Le-Cre* hemizygotes remain the most serious concern for the use of these mice. Although homozygous *P0-3.9GFPCre* mice appear healthy, fertile, and have phenotypically normal lenses, the widespread non-ocular CRE expression in early development makes these mice a problematic substitute for the *Le-Cre* strain. There clearly exists a need to recapture the CRE expression pattern of *Le-Cre* mice without the associated ocular phenotypes. The avoidance of CRE expression in the pancreas and retina would also improve the gene deletion specificity offered by *Le-Cre* mice.

In principle, three mechanisms may explain the *Le-Cre*-induced ocular abnormalities. These include (1) insertional mutagenesis, (2) the titration of a transcription factor endogenously activating *Pax6* expression in the lens or surface ectoderm, and (3) CRE or GFP toxicity. The similar phenotypic abnormalities occurring in two independently derived transgenic founder lines with different transgene integration sites exclude the insertional mutation mechanism. Others have suggested that multiple copies of the *Pax6* P0 promoter might effectively reduce transcription factors available to activate *Pax6* in the developing lens [30]. In fact, overexpression of *Pax6* suppressed ocular phenotypes associated with *Le-Cre* hemizygotes on a *CBA*-enriched genetic background [30]. However, a reduction in *Pax6* or other essential lens transcription factors should result in decreased expression of dependent transcripts. RNA-Seq analysis of *Le-Cre* hemizygous lenses failed to reveal any significant reduction of *Pax6* transcripts, PAX6-responsive transcripts, or transcripts of any other genes associated with lens development. The reasonably few transcriptional changes seen in hemizygous *Le-Cre* lenses include several genes associated with apoptosis and growth arrest. Given these observations, we suggest that the independent ocular phenotypes observed in *Le-Cre* transgenic mice result from CRE and/or GFP toxicity.

Numerous studies have shown that high-level, continuous expression of Cre recombinase can induce genomic instability and/or cell death. Male sterility in transgenic mice where the *Prm1* promoter drove CRE expression resulted from extensive chromosomal rearrangements in developing spermatids subsequent to the completion of meiosis II [37]. This phenotype exhibited complete penetrance in males and male offspring from five independent transgenic founders and required CRE enzyme activity. Likewise, CRE expression driven by the *surfactant protein C* promoter induces apoptosis in lung epithelial cells [46], and CRE expression from the *αMyHC* promoter leads to cardiotoxicity in transgenic mice [34]. CRE expression in keratinocytes, driven by either the *Keratin 5* or *Keratin 14* promoter, induces genomic instability, activation of p53, and cell cycle defects leading to frequent endomitosis and tetraploidy [33]. Although most transgenic studies attempt to direct CRE expression with tissue-specific regulatory sequences, transgenic mice often express CRE in secondary, unintended locations making CRE-dependent physiological phenotypes difficult to interpret [35]. Strikingly, the induction of CRE expression alone effectively led to apoptotic lymphoma regression in p53 deficient mice, demonstrating that CRE-induced apoptosis does not require p53 activity [47]. The toxic effects of CRE in transgenic mice appear to result from illegitimate recombination and/or DNA damage, initiated by CRE enzymatic activity at pseudo-LoxP sites, which occur at a frequency of 1.2 per megabase in the mouse genome [48].

In addition to CRE, the *Le-Cre* mice express GFP via an internal ribosome entry site (IRES) within the transgene construct [9] and GFP expression can also lead to toxicity. The most striking evidence for GFP-induced physiological phenotypes comes from studies of GFP expression in muscles [49–52]. Of four transgenic mouse lines made with GFP driven by the *αMyHC* promoter, mice from the two highest expressing lines developed lethal dilated cardiomyopathy [50]. GFP also interacts with the actin-binding site of myosin and interferes with muscle contraction [51, 52]. GFP expression can also lead to the activation of the immune system and increased oxidative stress [53]. However, ubiquitous GFP expression in many different transgenic lines without reported ocular phenotypes suggests that CRE expression is a more likely cause for the observed ocular defects in *Le-Cre* mice.

If the transgene-induced ocular phenotypes seen in *Le-Cre* mice result from toxicity, why are the other transgenic mice that express CRE in the lens apparently free from these abnormalities? The *P0-3.9GFPCre* mice also express both CRE and GFP in the lens, but our analysis demonstrates that this line expresses a lower level of CRE expression, both at the transcript and protein level, than the *Le-Cre* mice. Curiously, homozygous *MLR10* mice display none of the ocular abnormalities seen in virtually all homozygous *Le-Cre* mice. Although the *MLR10* mice do not express GFP, they exhibit higher CRE transcript expression in both the lens epithelium and lens fiber cells at birth than either *Le-Cre* or *P0-3.9GFPCre* mice (Fig. 8b). However, at E15, CRE protein appeared nearly absent from the lens epithelium in homozygous *MLR10* mice (Fig. 6). This incongruity could result from inefficient translation of CRE transcripts in the lens epithelium, a perinatal increase in lens epithelial CRE expression, or contamination of the lens epithelial RNA sample with differentiating secondary fiber cell RNA in *MLR10* transgenic mice. It is also possible that high expression of CRE protein at the lens placode stage has a uniquely deleterious effect on subsequent lens development, thus sparing *MLR10* mice where CRE expression initiates later. In any case, it appears that continuous, high-level expression of CRE from the placode stage and in the lens epithelium may compromise lens development.

It is possible that an efficient system to deliver transient, drug-inducible CRE to lens cell nuclei may avoid the ocular phenotypes seen in the *Le-Cre* mice. In particular, the CRE-ER system where CRE remains in the cytoplasm in the absence of tamoxifen ligand [54] represents a potential to limit the ability of CRE to induce DNA damage. Shi and Bassnett demonstrated that tamoxifen can induce postnatal CRE-mediated recombination in the lens using both a widely expressing CRE-ER transgene and GFP CRE reporter transgene [55], but this strategy only demonstrated mosaic recombination in the lens epithelium. It remains possible that an appropriate lens-directed CRE-ER transgene could lead to widespread tamoxifen-inducible recombination in either the postnatal lens or in the lens placode dependent on the timing of drug administration. Other inducible systems may also work in the developing lens and avoid continuous CRE expression in the lens epithelium. In these cases, investigators will also have to control for drug administration in the analysis of resulting phenotypes.

## Conclusion

Choosing the best option for CRE delivery in studies of the lens requires careful consideration of both the timing and the pattern of CRE expression. Here, we have provided an analysis based on the previously produced CRE transgenic mice that express CRE in the lens. While each of the existent CRE transgenic lines have unique properties that may be advantageous for a particular experiment, it remains imperative that any studies using these mice contain the appropriate controls. These controls must include the analysis of animals expressing CRE in the absence of targeted, floxed alleles. The omission of this control makes it difficult or impossible to determine if the observed phenotype truly represents the loss of a targeted gene or an independent, CRE-mediated effect.

## Methods

### Experimental animals

Inbred *FVB/N* mice were purchased from Harlan Sprague Dawley. Mice were bred and maintained in the Miami University Animal Facility. All experimental manipulations received approval from the Miami University Institutional Care and Use Committee and conformed to the ARVO Statement for the Use of Animals in Ophthalmic and Vision Research. *Le-Cre (Tg(Pax6-cre, GFP*)*1Pgr)* transgenic mice, on the original *FVB/N* background [9], were obtained from Dr. Ruth Ashery-Pandan (Department of Human Molecular Genetics and Biochemistry, Sackler Faculty of Medicine, Tel Aviv University). *P0-3.9GFPCre* (*Tg(Pax6-GFP/cre*)*1Rilm*) transgenic mice [12] were originally produced by Dr. Richard Maas (Department of Medicine, Division of Genetics, Harvard University, Boston, MA), and obtained on an *FVB/N* inbred background from Dr. Salil Lachke (Department of Biological Sciences, University of Delaware). *MLR10* mice were produced on an *FVB/N* background as described [8] and the ROSA26 Cre reporter strain, *Gt(ROSA)26Sor*^*tm1Sor*^, was obtained from The Jackson Laboratory (Bar Harbor, ME). Hemizygous *Le-Cre*, *P0-3.9GFPCre*, and *MLR10* were generated by crossing homozygous mice with either *FVB/N* or ROSA26 CRE reporter strains.

### Evaluation of Cre transgene expression

Timed pregnancies were obtained from *Le-Cre* and *P0-3.9GFPCre* mice crossed with the ROSA26 Cre reporter strain [32], with noon of the day of vaginal plug observation considered E0.5 days of embryogenesis. Embryos from embryonic days 7.5, 8.5, 9.5, 10.5, 12.5, 15.5, and the day of birth (P0) were collected for evaluation. Embryos were visualized for GFP expression (co-expressed with CRE in both the *Le-Cre* and *P0-3.9GFPCre* mice) under a SteREO Discovery.V12 microscope (Zeiss). A Zeiss AxioCam MRc5 with AxioVison Extended Focus module was used to obtain images with increased focus depth. To visualize LacZ expression, embryos were lightly fixed with phosphate buffered 4% paraformaldehyde (Fisher chemical, Germany Cat. No. O4042), washed three times in phosphate buffered saline (PBS), and incubated in the dark overnight at room temperature in an X-Gal staining solution (40 mg/ml X-Gal, 50 mM potassium ferrocyanide, 50 mM potassium ferricyanide, 10% deoxycholate/20%NP4-, 1 M MgCl_2_ in 1xPBS). The next day, the samples were rinsed in PBS and post-fixed with 10% neutral buffered formalin (Thermo Scientific, MI, USA Cat. No. 5725) overnight at room temperature while protected from light. Following three washes with PBS, embryos were either photographed in whole mount or incubated in the dark at 4 °C in increasing sucrose concentrations from 7.5% to 30% sucrose for 12 h each step. Tissues were then embedded in a 30% sucrose/OCT mixture at 1:2 ratio and frozen and cryosectioned at 12 μm thickness. Embryonic heads were sectioned anterior-posterior in a horizontal plane to include the cornea, lens, and retina. To visualize internal LacZ expression, frozen sections were stained with X-Gal and post-fixed with paraformaldehyde before mounting.

### FISH analysis

Bone marrow was aspirated from the femur of transgenic and wild-type mice and grown overnight in RPMI media supplemented with 15% FBS, (Gibco BRL). Subsequently, cells were harvested according to standard cytogenetics methods and stored in Carnoy fixative (3:1, methanol:glacial acetic acid) at − 20 °C. The BAC probes were labeled with Spectrum Orange or Spectrum Green using a nick translation kit (Vysis, Downer Groves, IL) according to the manufacturer‘s recommendations. The reaction was carried out for 8 h at 15 °C and stopped by heating the sample to 70 °C for 10 min. FISH was performed on freshly prepared slides. The DNA was combined with mouse Cot-1 DNA (Gibco BRL), ethanol precipitated, resuspended in Hybridization Buffer (Sigma, St. Louis, MO), and denatured. The probes were applied to previously denatured slides. The hybridization was carried out overnight at 37 °C and slides were washed in 2xSSC/0.1% NP-40 for 5 min at 42 °C. The signals were viewed with fluorescence microscope (Zeiss Axioscope 40) equipped with appropriate filters, and analyzed with the Applied Imaging System. Physical localization of the *Le-Cre* transgene to chromosome 16 was confirmed using probes derived from both Cre recombinase and the NOD/Mrk Tac GSS BAC clone DN-4E11. FISH analysis of the RD6 fibroblasts utilized a digoxigenin-labeled probe for CRE and a biotin-labeled probe for BAC RP24-257H11. Hybridization signals were visualized with red and green fluorescently labeled antibodies for digoxigenin and biotin, respectively.

### Routine histology

P0 and P21 pups of homozygous and hemizygous *Le-Cre*, *P0-3.9GFPCre*, and *MLR10* were collected, genotyped, imaged, fixed with 10% neutral buffered formalin, processed to paraffin embedded, and sectioned at 7 μm thickness. The samples were sectioned in an anterior-posterior plane to include the cornea, lens, and retina. To avoid the lenses shattering, the wax block was kept wet during sectioning. Histological features were compared using standard hematoxylin and eosin (H & E) staining methods.

### Evaluating CRE protein expression through immunohistochemistry

E15.5 embryo heads were collected and fixed in 10% neutral buffered formalin at room temperature overnight. Standard protocols were used to process and embed tissues in paraffin wax. Paraffin blocks were sectioned at 5 μm onto slides. The slides were washed in xylene and alcohol to remove paraffin and rehydrate the tissue. Then, the protein antigens on the sections were retrieved as previously described [5]. The slides were blocked with 10% normal goat serum in PBST. The primary antibody for CRE was purchased from Cell signaling 12830 (USA) and used at a 1:100 dilution. The primary CRE antibody was detected using a goat anti-rabbit IgG secondary antibody conjugated to Alexa Fluor 488 fluorescent probe (1:500 dilution). The sections were counterstained with DAPI (Vector Labs H-1200, Burlingame, CA, USA).

### Evaluating CRE protein expression through western blot

Lenses were dissected from newborn mice and homogenized in RIPA buffer (50 mM Tris HCl pH 8, 150 nM NaCl, 1% NP40, 0.5% sodium deoxycholate) with protease inhibitor (Sigma-Aldrich, St. Louis, MO, USA). Equal amount of protein lysates were separated by 1% SDS-contained 10% polyacrylamide gel electrophoresis (SDS-PAGE) then transferred to PVDF membrane. The blot was blocked in blocking solution containing 5% non-fat dry milk in 0.1% PBST for 2 h at room temperature. Then, the blots were incubated at 4 °C overnight with either at 1/3000 rabbit monoclonal anti-CRE antibody (Cell signaling 12830, Carlsbad, CA), or 1/1000 rabbit polyclonal anti-GAPDH antibody (Cell signaling 2118) diluted in blocking solution. The next day, the blots were washed with 0.1% PBST and then incubated with the goat anti-rabbit HRP-conjugated secondary antibodies (1:5000 in 0.1% TBST) for 1 h. The Clarity™ Western ECL Substrate (Bio-Rad 1705061, Hercules, CA) and resultant AutoRadiography film (Fisher NC9556985, USA) were used for detection and image development. Finally, quantification of the proteins of interest was done relative to GAPDH using Image J software.

### Evaluating CRE expression level through whole transcriptome Illumina sequencing

Newborn lenses from wild-type *FVB/N*, hemizygous *Le-Cre*, and hemizygous *P0-3.9GFPCre* mice were collected at P0 and pooled into three biological replicates, each containing six lenses from three mice. Total RNA, including mRNA, was extracted using the mirVana mRNA isolation kit (AM1560, ThermoFisher) according to the manufacturer’s recommendations. Total RNA samples with the RNA integrity number (RIN, Agilent 2100 Bioanalyzer) ≥ 8.0 were used to prepare a library of template molecules suitable for subsequent sequencing on an Illumina (St. Louis, MO) HiSeq platform. Polyadenylated RNA was purified from the total RNA samples using Oligo dT conjugated magnetic beads and prepared for single-end sequencing according to the Illumina TruSeq RNA Sample Preparation Kit v3. The resultant library was sequenced for 50 cycles using the TruSeq SBS kit on an Illumina HiSeq 2000 system at the Genomics and Sequencing Core Laboratory at the University of Cincinnati. Approximately 29 million single-end sequence reads of 51 bp per sample were generated, and these sequences were mapped back to the mouse genome assembly GRCm38 (mm10). Adapters and poor-quality regions were trimmed using Trimmomatic-0.36 software. Gene and isoform abundance was quantified using RSEM-1.3.0 software. Reads were mapped to the C57BL/6J reference genome using GSNAP software. Differential expression analysis was completed using DESeq2-1.10.1 software. For differential expression, we used a cutoff value of equal to or greater than 1.5-fold change with an adjusted *p* value ≤ 0.05.

### Real-time PCR

Lenses were manually removed from the eye at P0 from hemizygous *Le-Cre*, *P0-3.9GFPCre*, and *MLR10* transgenic lines. Whole lenses, epithelium, and fiber cells were separately pooled into three biological replicates each containing six lenses or six fractions from three mice for each transgenic line. For the epithelial cell fraction and fiber cell fraction, lenses were dissected following the procedure previously described [56]. Total RNA was isolated using the Mini Total RNA Kit for Tissue (IBI Scientific IB47302, Peosta, IA, USA) and reverse-transcribed using the SuperScript IV First-Strand Synthesis System (Invitrogen 18091050, Carlsbad, CA, USA) according to the manufacturer’s recommendations. Each biological cDNA replicate was amplified by qPCR three times. Real-time quantitative PCR analysis was performed using the SYBR-Green GoTaq qPCR Master Mix (Promega A6001, Madison, WI, USA) on a CFX Connect – 96 well system (Bio Rad). The expression levels of individual genes were normalized to GAPDH mRNA levels and analyzed using the delta-delta Ct method (Applied Biosystems) with significant differences revealed by a two-tailed Student’s *t* test. The primer sequences used in the real-time PCR assays for murine GAPDH are GACGTGCCGCCTGGAGAAAC forward and AGCCCAAGATGCCCTTCAGT reverse. The primer sequences used for CRE are CCTGTTTTGCACGTTCACCG forward and ATGCTTCTGTCCGTTTGCCG reverse.

## Additional file

Additional file 1: **Figure S1.** FISH analysis using a probe for CRE labeled in red and a mouse BAC probe (RP24-257H11) labeled in green. Mitotic chromosome spreads from fibroblasts established from the last living homozygous transgenic mouse from the second founder (RD6) created with the identical DNA construct that established the Le-Cre transgenic line. An idiogram of mouse chromosome 10 with the location of transgene insertion (green arrowhead) is shown on the idiogram at the left of the FISH image. White arrows point to the overlapping FISH signals on each of the copies of chromosome 10. The idiogram was taken from the Idiogram Album by David Adler © 1994. (PDF 235 kb)

## Abbreviations

AER: Apical ectodermal ridge
ce: Corneal epithelium
CRE: Cre recombinase
E9.0: Embryonic day 9.0
FISH: Fluorescence in situ hybridization
floxed: LoxP-flanked
GFP: Green fluorescent protein
GO: Gene ontology terms
H & E: Hematoxylin and eosin
IRES: Internal ribosome entry site
le: Lens
*Le-Cre*^*Hemi*^: Hemizygous *Le-Cre*
*Le-Cre*^*Homo*^: Homozygous *Le-Cre*
lf: Lens fiber cells
*mαA-Cre*: Mouse αA-crystallin promoter transgenic CRE
Nav1.9: *Scn11A* encodes a voltage-gated sodium channel protein
nr: Neural retina
P0: Embryonic day 0
P21: 3 weeks after birth
RPE: Retinal pigmented epithelium
TAg: SV40 large tumor antigen
