# Supplementary material for: Considerations for the use of Cre recombinase for conditional gene deletion in the mouse lens
Source: Hum Genomics. 2019 Feb 15;13:10. doi: 10.1186/s40246-019-0192-8 (PMC6377743; doi:10.1186/s40246-019-0192-8)
Supplement: Supplementary file 1 — Figure S1. FISH analysis using a probe for CRE labeled in red and a mouse BAC probe (RP24-257H11) labeled in green. Mitotic chromosome spreads from fibroblasts established from the last living homozygous transgenic mouse from the second founder (RD6) created with the identical DNA construct that established the Le-Cre transgenic line. An idiogram of mouse chromosome 10 with the location of transgene insertion (green arrowhead) is shown on the idiogram at the left of the FISH image. White arrows point to the overlapping FISH signals on each of the copies of chromosome 10. The idiogram was taken from the Idiogram Album by David Adler © 1994. (PDF 235 kb) [file 40246_2019_192_MOESM1_ESM.pdf]

Supplemental Figure 1

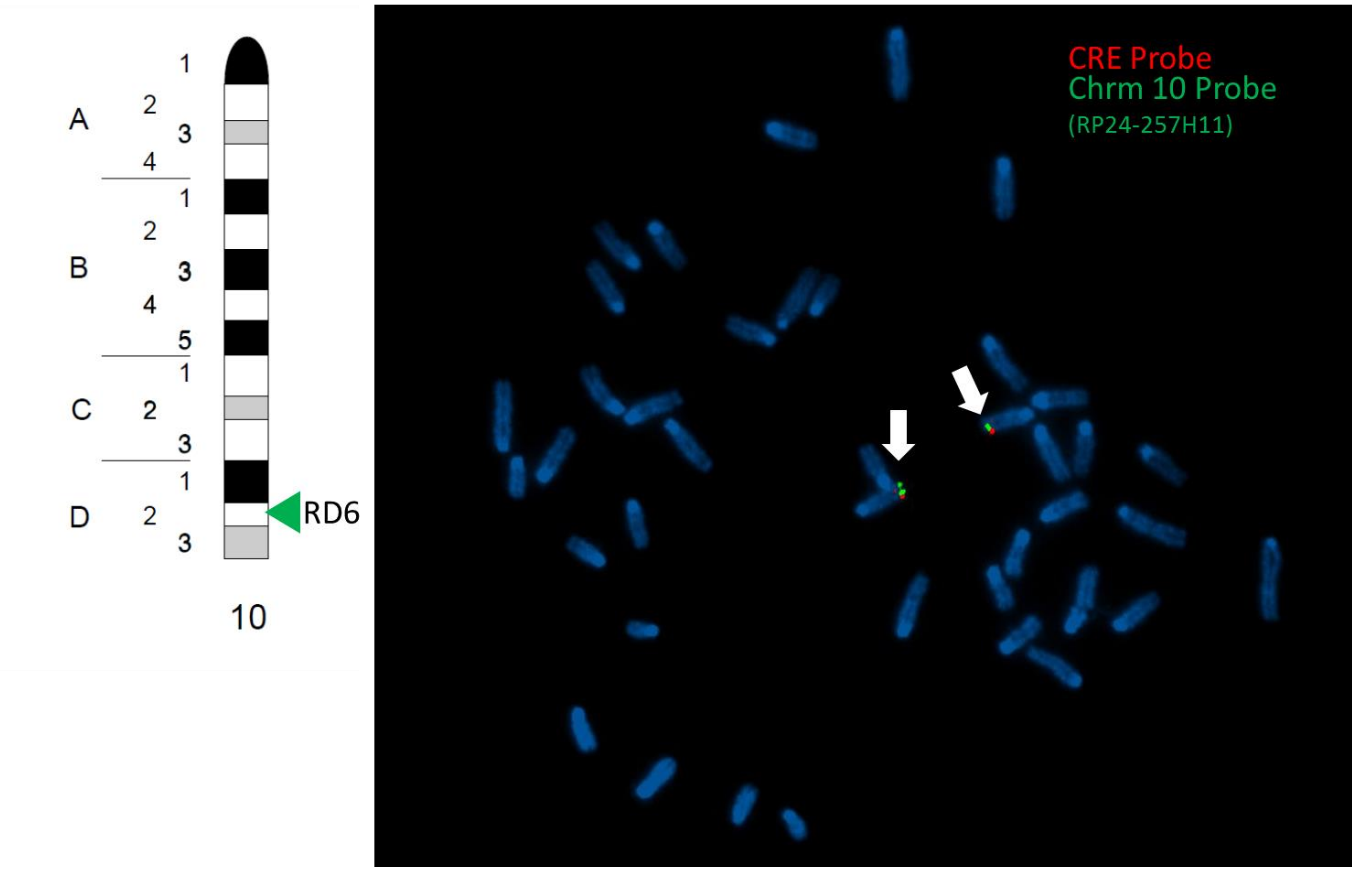

Supplemental Figure 1: FISH analysis using a probe for CRE labeled in red and a mouse BAC probe (RP24-257H11) labeled in green. Mitotic chromosome spreads from fibroblasts established from the last living homozygous transgenic mouse from the second founder (RD6) created with the identical DNA construct that established the Le-Cre transgenic line. An idiogram of mouse chromosome 10 with the location of transgene insertion (green arrowhead) is shown on the idiogram at the left of the FISH image. White arrows point to the overlapping FISH signals on each of the copies of chromosome 10. The idiogram was taken from the Idiogram Album by David Adler © 1994.
